# Oral liposarcoma in elderly: Case report and literature analysis

**DOI:** 10.1097/MD.0000000000018985

**Published:** 2020-02-07

**Authors:** Keiichi Ohta, Hitoshi Yoshimura, Shinpei Matsuda, Yoshiaki Imamura, Kazuo Sano

**Affiliations:** a Department of Dentistry and Oral Surgery, Unit of Sensory and Locomotor Medicine, Division of Medicine, Faculty of Medical Sciences; b Division of Surgical Pathology, University of Fukui Hospital, Fukui, Japan.

**Keywords:** adults aged 80 over, biopsy, buccal mucosa, elderly, liposarcoma

## Abstract

**Rationale:**

Oral liposarcoma is an extremely rare lesion that is often clinically misdiagnosed as a benign lesion because of its asymptomatic and indolent clinical course. we report a case of atypical lipomatous tumor/well-differentiated liposarcoma (ALT/WDL) of buccal mucosa, provisionally diagnosed as lipoma.

**Patient concerns:**

A 97-year-old female was referred to dentistry and oral surgery department with an asymptomatic mass on the right buccal mucosa which had been present for an unknown period of time.

**Diagnosis:**

Magnetic resonance imaging demonstrated a well-circumscribed lesion at the right buccal mucosa, and a lipoma was suspected.

**Interventions:**

Surgical removal was performed, and a diagnosis of ALT/WDL was made. She and her family refused additional treatment due to her age.

**Outcomes:**

At the 10 months follow-up, the patient remained free of disease.

**Lessons:**

The indolent clinical course and small size of oral liposarcoma can lead to provisional clinical diagnosis of benign lesion.

## 1 Introduction

Liposarcoma is the most common soft tissue sarcoma of adults, and makes up 15% to 25% of all sarcomas. It usually occurs in the deep soft tissues of the lower extremities and retroperitoneum of middle-aged adults.^[1]^ In the head and neck region, liposarcoma is rare, and is found in up to 9% of cases.^[2]^ Oral liposarcoma is even more rare, occurring mainly in the buccal mucosa and tongue of middle-aged adults with a male predominance.^[3–7]^ Liposarcoma is divided into 4 subtypes: atypical lipomatous tumor/well-differentiated liposarcoma (ALT/WDL), myxoid liposarcoma, pleomorphic liposarcoma, and dedifferentiated liposarcoma. ALT/WDL is categorized as intermediate (locally aggressive) adipocytic tumors and is the most common subtype of liposarcoma, making up 30% to 40% of all liposarcomas.^[1]^ In the oral region, ALT/WDL and myxoid liposarcoma are the predominant subtypes.^[5,7–10]^ These tumors have a tendency of local recurrence, but distant metastasis rarely occurs unless these tumors become dedifferentiated.^[2,11]^ Oral ALT/WDL may often be misdiagnosed as a non-malignant lesion because of its asymptomatic condition of slow-growing, painless, and circumscribed submucosal mass which may be present for several months or years before the diagnosis.^[6,7]^ In addition, insufficient treatment may be given due to its rarity of incidence and complex histopathological features.^[12–14]^ Herein, we report a case of ALT/WDL of buccal mucosa in a 97-year-old female, provisionally diagnosed as lipoma. We also review the English-language literature to investigate the association of preoperative diagnosis with treatment and prognosis of oral ALT/WDL.

## 2 Case report

**Figure 1:**
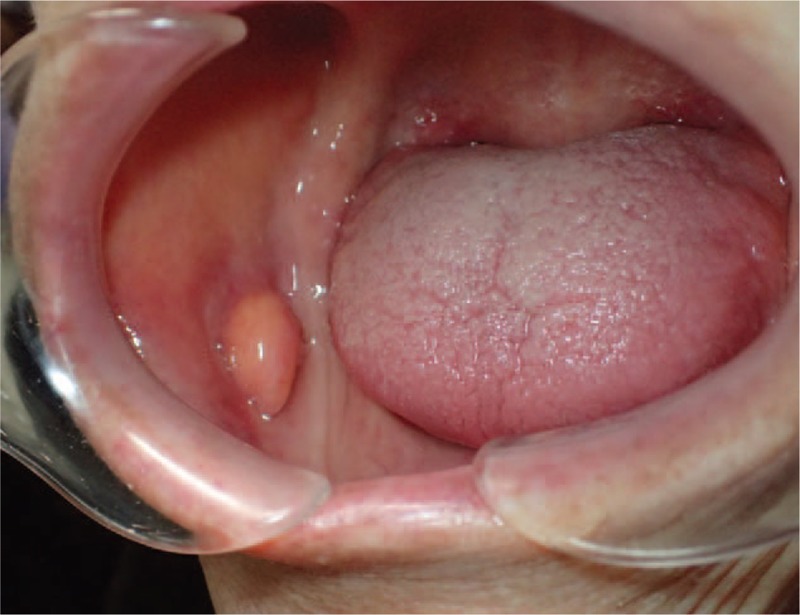
Intraoral examination showing a 20 mm, elastic soft, painless, smooth, and well-circumscribed yellow mass covered by normal mucosa on the right buccal mucosa.

**Figure 2:**
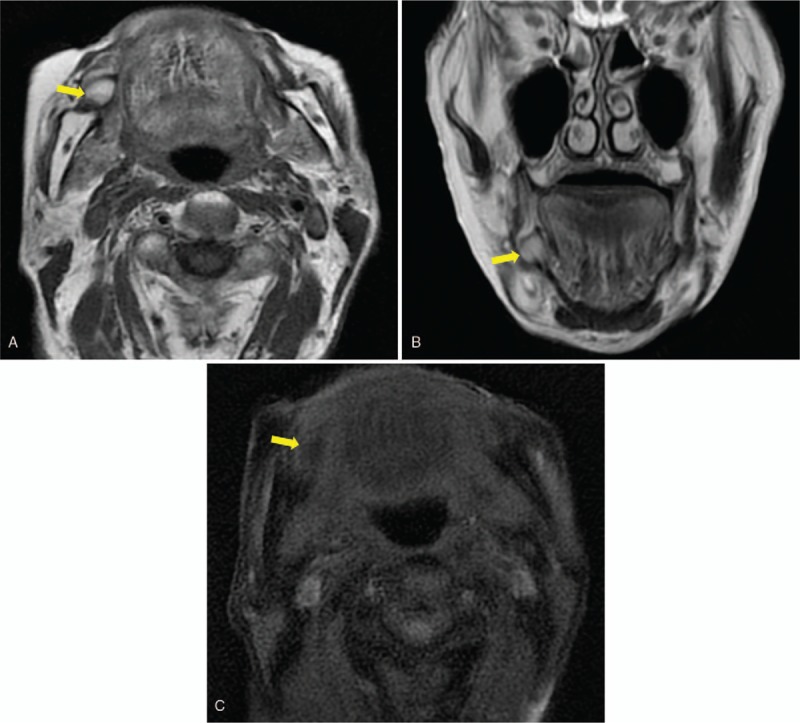
Axial and coronal MRI images showing a well-circumscribed lesion at the right buccal mucosa. The mass revealed high-signals in both T1-weighted (A, arrow) and T2-weighted images (B, arrow) and low-signals in fat-suppression T1-weighted images (C, arrow). MRI = magnetic resonance imaging.

**Figure 3:**
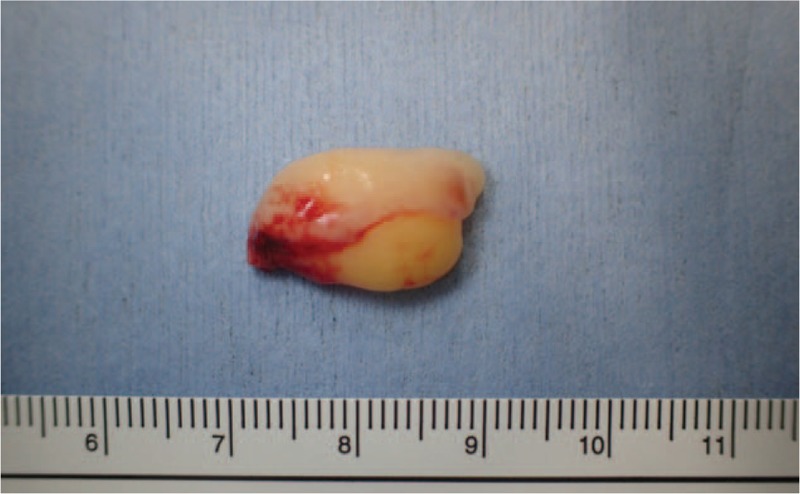
The removed specmen showing a pale yellow, non capsulated mass.

**Figure 4:**
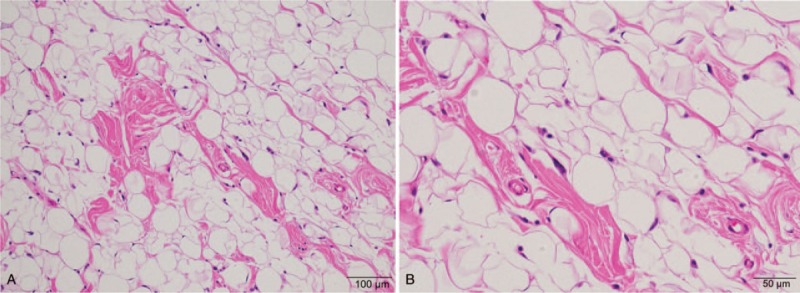
Histopathological findings showing proliferation of almost uniform sized adipocytes with hyperchromatic stromal cells in the fibrous connective tissue, hematoxylin and eosin stain; magnification, (A) × 200, (B) × 400).

**Figure 5:**
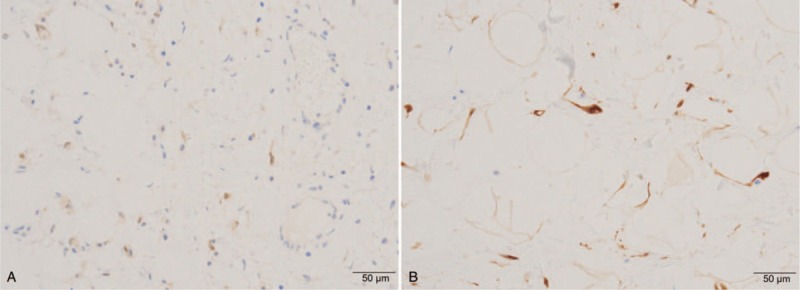
Immunohistochemical examination showing positive results for p16 (A, magnification, × 400) and CDK4 (B, magnification, × 400). CDK4 = cyclin dependent kinase 4.

A 97-year-old female was referred to our department with an asymptomatic mass on the right buccal mucosa which had been present for an unknown period of time. The patient's past medical history revealed dementia, type 2 diabetes mellitus, and neurogenic bladder. Intraoral examination showed a 20 mm, elastic soft, painless, smooth, and well-circumscribed yellow mass covered by normal mucosa on the right buccal mucosa (Fig. 1). Extraoral examination showed no cervical lymphadenopathy. Magnetic resonance imaging (MRI) demonstrated a well-circumscribed lesion at the right buccal mucosa. The mass revealed high-signals in both T1-weighted and T2-weighted images and low-signals in fat-suppression T1-weighted images (Fig. 2). With a provisional diagnosis of a lipoma, the patient underwent surgical removal of the mass under local anesthesia. The removed specimen revealed a pale yellow, non-capsulated mass (Fig. 3). Histopathological examination revealed proliferation of almost uniform-sized adipocytes with hyperchromatic stromal cells in the fibrous connective tissue (Fig. 4). Immunohistochemical examination revealed positive results for p16 and cyclin dependent kinase (CDK4) (Fig. 5), and a few cells revealed weak positivity for murine double minutes 2 (MDM2). Based on the findings, a diagnosis of ALT/WDL (lipoma-like) was made. The patient and her family refused to take additional treatment due to her age, and we did not perform additional surgical treatment or postoperative radiotherapy. ^18^F-fluorodeoxyglucose positron emission tomography/computed tomography (FDG-PET/CT) scans at 1 month and 4 months after surgery revealed no evidence of local recurrence and distant metastasis. At the 10 months follow-up, the patient remained free of disease.

## 3 Discussion

We searched the English language literature published between 1979 and 2018 using PubMed and Google Scholar. We identified 120 cases of oral liposarcoma (Table 1).^[3–6,8–10,12–66]^ The most common subtypes of liposarcoma was ALT/WDL (60.8%), followed by myxoid liposarcoma (17.5%), dedifferentiated liposarcoma (6.7%), and pleomorphic liposarcoma (3.3%). The predominance of ALT/ WDL and myxoid liposarcoma in the oral region was consistent with the previously reported studies.^[5,8,9]^ The mean age is 50 years (from 6 months to 97 years). According to the available data in 114 cases, there was no particular sex predilection (male-to-female ratio 1:1.04). The most common location of oral liposarcoma was the tongue (40.7%), followed by cheek/buccal mucosa (31.4%), gingiva (7.6%), palate (5.9%), oral floor (5.1%), lip (4.2%), and other areas (5.1%). The most predominant site for oral liposarcoma was inconsistent among previous studies - either the tongue or cheek/buccal mucosa.^[4–6,8,9]^ Most of cases appeared as a circumscribed, indolent, asymptomatic, firm or elastic hard or soft submucosal mass. The mean size of tumor was 29.6 mm (from 3 mm to 120 mm), and the mean duration was 25.5 months (from 0.3 to 168 months). In our review, ulcer formation was observed only in one case, and pain and bone resorption were observed in 4 and 5 cases before initial treatment, respectively. Distant metastasis or lymph node metastasis was not observed. One case of dedifferentiated liposarcoma metastatic to the gingiva was reported.^[56]^ Recurrence was reported in twenty cases, and 7 patients died of disease. The indolent clinical course, small size, and poor clinical and imaging findings of oral liposarcoma may lead to provisional clinical diagnosis of benign lesion or difficulty to suspect malignancy.

**Table 1:**
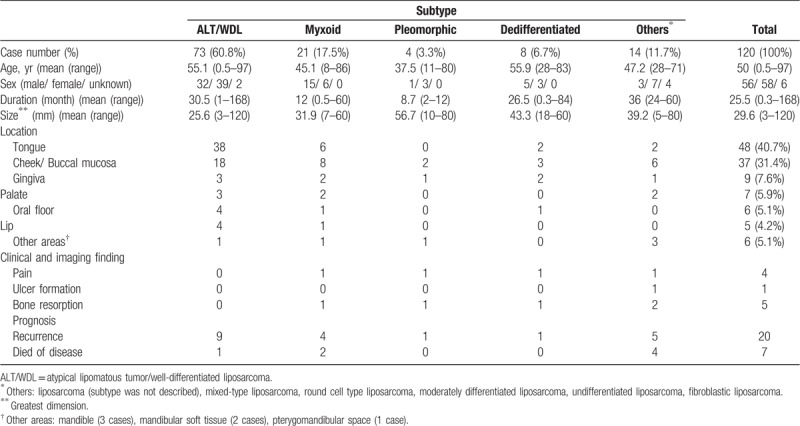
Reported cases of oral liposarcoma in the English literature.

Since 1979, 6 cases of oral liposarcoma in patients aged over 80 years have been reported in the English language literature (Table 2).^[4,24,26,42,47]^ There was no particular sex prevalence (4 males and 3 females), and the predominant site was the tongue (3 cases) and cheek/buccal mucosa (3 cases). The most reported subtype of liposarcoma was ALT/WDL. These trends resembled the 120 cases of oral liposarcoma mentioned in Table 1. The size of tumor was relatively small (the mean size of tumor was 25.7 mm) in the patients over 80 years and no cases were operated on aggressively. Although we know of only 1 case of myxoid liposarcoma of the medial thigh in a 91-year-old male,^[67]^ to the best of our knowledge, our patient was the oldest case of liposarcoma known with disease-entity.

**Table 2:**
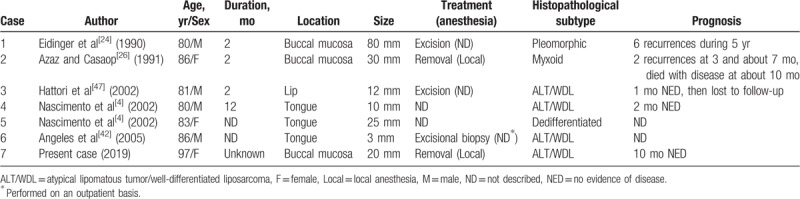
Oral liposarcoma in elderly patients over 80 yr old in the English literature.

MRI and computed tomography (CT) can be very useful tools for the evaluation of soft tissue masses because fat can be easily identified.^[68]^ Because of its superior soft tissue contrast resolution, MRI is the best imaging tool for evaluating the extent and internal structure of soft tissue tumors.^[69]^ Characteristics which may assist in distinguishing ALT/WDL include age (over 60 years), size of the lesion (over 10 cm), presence of enhancing thickened septa (over 2 mm), the presence of non-adipose lesions, and lower proportion of fat (under 25% of tumor volume).^[69]^ These image findings may help differential diagnosis. However, both ALT/WDL and lipoma consist of more than 75% fat, and the features of ALT/WDL and benign lipomatous tumors extensively overlap.^[69]^ Therefore, the differentiation between lipoma and ALT/WDL using CT or MRI, especially in a small mass in the oral region like our case, is considered very difficult.^[68,69]^ This is why histopathological examination is mandatory.^[7]^ Besides, we did not use contrast medium considering the patient's burden on the kidney, which made preoperative diagnosis more challenging. We must also note the previous report of ALT/WDL of the gingiva that did not suspect liposarcoma on preoperative gadolinium-enhanced MRI.^[14]^ Compared with ALT/WDL, other histologic subtypes of liposarcoma have a higher quota of non-adipose elements.^[69]^ When a presumed lipoma has any complexity, liposarcoma should be taken into account.^[68]^ Recently, intraoral ultrasonography has been utilized for preoperative diagnosis.^[62,70]^ Sugawara et al reported that intraoral ultrasonographic findings reflected the histological findings in the cases of tongue mass lesions.^[62]^ They showed the intraoral ultrasonographic finding of liposarcoma (subtype was not described) in the tongue; the tumor was a hypoechoic homogenous mass with an irregular border, and blood flow was observed in the center of the lesion. FDG-PET/CT also offers good sensitivity for assessment of sarcoma, including liposarcoma, and detection of their recurrence.^[71]^ Suzuki et al demonstrated that FDG-PET is useful for differentiating between benign lipomatous tumors and ALT/WDL in the extremities.^[72]^ Our patient was twice evaluated for local recurrence or distant metastasis by FDG-PET/CT and she remained free of disease during 10 months of follow-up.

ALT/WDL is subdivided into 3 subtypes: lipoma-like, sclerosing, and inflammatory.^[1]^ The differentiation between lipoma-like and sclerosing type is often difficult and has limited practical importance since ALT/WDL has characteristics of both lipoma-like and sclerosing type.^[1]^ Lipoma-like ALT/WDL typically shows a predominance of mature fat with a variety of spindled cells with hyperchromatic nuclei and multivacuolated lipoblasts. However, it is challenging to differentiate lipoblasts from their histological mimics.^[73]^ Besides, lipoblasts are hardly identified in ALT/WDL and may exist in some benign lipogenic tumors. Recently, diagnosis is based more on the identification of atypical stromal cells rather than the identification of lipoblasts.^[1]^ Differential diagnosis of ALT/WDL includes various neoplastic and non-neoplastic lesions, such as lipoma, intramuscular lipoma, spindle cell lipoma, pleomorphic lipoma, chondroid lipoma, myolipoma, angiolipoma, angiomyolipoma, lipoma with fat necrosis, lipoma with Lochkern (adipocyte with intranuclear vacuoles), and atrophy of fat.^[1,6,73]^ For differentiation of ALT/WDL from various benign adipocytic lesions, additional immunohistochemical analyses are often required.^[74]^ ALT/WDL and dedifferentiated liposarcoma are characterized by giant marker and/or ring chromosomes. The giant marker and ring chromosomes comprise an amplified sequence of the 12q13-15 region, leading to the amplification of several genes.^[1]^ In ALT/WDL and dedifferentiated liposarcoma, both MDM2 and CDK4 are regularly amplified and expressed,^[75,76]^ resulting in overexpressed proteins which can be detected by immunohistochemical examination.^[77]^ MDM2 binds and inhibits tumor suppressor p53, thus decreasing apoptosis. On the other hand, CDK4 phosphorylates the RB (retinoblastoma protein), which inhibits its interaction with E2F transcription factor, allowing it to escape the G1-S checkpoint.^[1]^ However, we must keep in mind that MDM2 expression may appear not only in tumor cells, but also in histocytes, which is observed in lipoma with degenerative changes.^[74]^ Recent studies showed the utility of a combination of MDM2, CDK4, and p16 (cyclin-dependent kinase inhibitor 2A) as useful markers for detecting ALT/WDL and dedifferentiated liposarcoma.^[77]^ p16 binds CDK4 and inhibits cell cycle progression and its level is supposed to be correlated with the level of MDM2 and CDK4.^[78]^ These immunohistochemical examinations help provide a more accurate differential diagnosis, especially when a molecular diagnosis, which uses MDM2 amplification assessed by fluorescence in situ hybridization, is not available.^[77]^ Thway et al found that expression of MDM2 tended to be weak but that of CDK4 and p16 was mostly moderate to strong in ALT/WDL,^[77]^ which was also observed in our case.

The treatment for head and neck liposarcoma is mainly surgery with negative margins.^[7,79]^ Neck dissection is not necessary unless there is concrete evidence of lymph node metastasis, since lymph node metastasis is so rare.^[80]^ McCulloch et al reported that incomplete excision was associated with 80% of local recurrence or distant metastasis in head and neck liposarcoma, and in those, 62.5% of the cases were pleomorphic or round cell (now referred as high grade myxoid type) liposarcoma.^[81]^ In the head and neck region, a radical surgical approach can involve neurovascular and important anatomical structures and lead to functional and esthetic disorders. Some authors reported that conservative surgical excision with close follow-up should be preferred for head and neck ALT/WDL because of its clinicopathologic features and indolent biological behavior.^[14,61,82]^ Postoperative radiotherapy should be considered in cases of large tumors, those in a deep anatomical location, high grade tumors, positive margins and local extension.^[79]^ However, due to the absence of systemic studies comparing the outcomes of treatment with or without radiotherapy, the efficacy of radiotherapy has been obscure.^[82]^ As for chemotherapy, myxoid liposarcoma is relatively chemosensitive compared with other subtypes of liposarcoma.^[83,84]^

For head and neck liposarcoma, the factors for better prognosis are associated with small size, the superficial site, and the histopathological predominance of the ALT/WDL variant. In oral liposarcoma, a maximum diameter not greater than 50 mm or 36 mm is considered a favorable prognostic factor.^[5,7,38]^ The prognosis is poor when ALT/WDL occurs in the deep-seated area. ALT/WDL and myxoid liposarcomas have a better prognosis than the other histologic subtypes. On the other hand, pleomorphic, round cell (now referred as high grade myxoid type) and dedifferentiated liposarcomas show a higher incidence of recurrence and metastasis. Davis et al investigated 30 cases of liposarcomas in the head and neck region and reported crude disease-specific survival was 100% for ALT/WDL and myxoid liposarcoma, but 60% for round cell and 45% for pleomorphic liposarcoma.^[79]^ This finding suggests that discrimination of the histological subtype of liposarcoma is pivotally important in predicting the prognosis.^[66]^

To gain a better understanding of the clinical characteristics and management of oral liposarcoma, reported cases were reviewed (Table 3). Since 1979, 45 well-documented cases have been reported in the English language literature.^[3,5,6,8,9,13–17,19,20,25,29,30,32,35,38,39,41–43,47,50,52,54,57,58,60,64,65]^ The reported data included presence of preoperative biopsy, preoperative diagnosis, treatment and prognosis. Biopsy was undertaken in 22 cases, and 77.3% of cases were diagnosed with ALT/WDL or liposarcoma, while 18.2% of the cases were misdiagnosed with benign lesion. Among the cases diagnosed correctly, excisional biopsy was performed in 70.6%, and re-excision was performed in 58.3%, while no additional treatments were carried out in 33.3%. No recurrence was observed in the cases with excisional biopsy. In the group diagnosed as benign lesion by biopsy, incisional and not described biopsy (1 case) were performed, and 50% of the cases (2 cases) were untreated, which led to recurrence. On the other hand, 23 cases were treated without biopsy, and conservative surgical treatment was performed in all cases (100%), with provisional clinical diagnosis of benign tumor in most cases. In those, additional treatment was undertaken in 13% after the definitive diagnosis. Recurrence occurred in 17.4% of the cases without biopsy. These data imply that, if possible, excisional biopsy is preferable for accurate diagnosis, which may assist in avoiding inadequate treatment. In this case, the tumor was small size, located on the superficial site, and diagnosed as ALT/WDL. These conditions suggest good prognosis can be expected. However, follow-up duration of this case is short and further close follow-up is mandatory because the patient was treated conservatively and the recurrence rate is not low. A literature review by Nimura et al which investigated 50 cases of head and neck dedifferentiated liposarcoma showed that preoperative biopsy by either incision or fine needle aspiration is not reliable for the diagnosis of dedifferentiated liposarcoma.^[63]^ In their study, dedifferentiated liposarcoma was diagnosed only in 23.1% of the cases by biopsy, and 46.2% of cases were misdiagnosed as benign lesions or failures. To overcome the difficulty of precise preoperative diagnosis, immunohistochemical examination of biopsy specimen may be a potential diagnostic tool.^[3,66]^

**Table 3:**
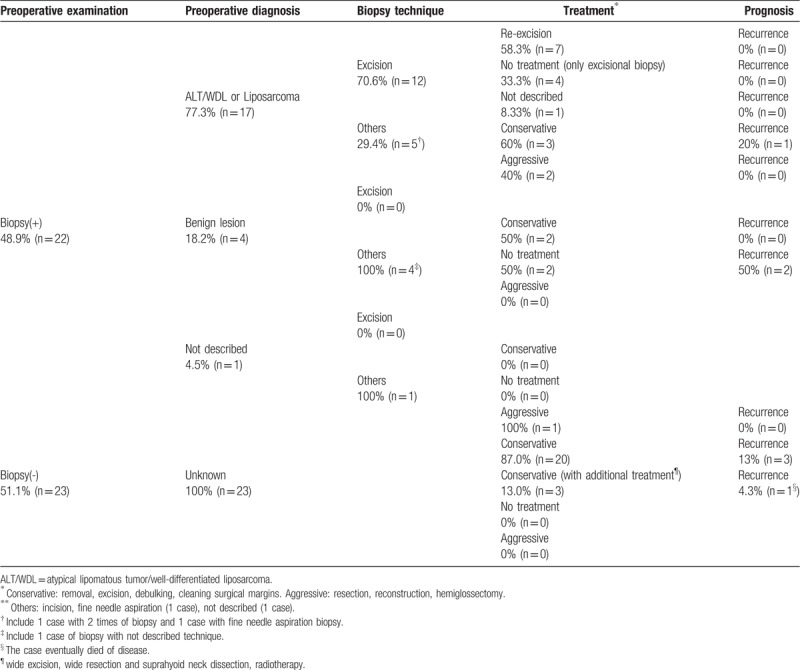
Association of preoperative diagnosis with treatment and prognosis in oral ALT/WDL.

## 4 Conclusion

In conclusion, we reported the oldest patient with oral liposarcoma. The indolent clinical course and small size of oral liposarcoma can lead to provisional clinical diagnosis of benign lesion. Excisional biopsy is preferable for accurate diagnosis, which may assist in avoiding inadequate treatment. When conservative surgical treatment is performed before the diagnosis of ALT/WDL, and there is difficulty selecting additional treatment, close follow-up is essential.

## Author contributions

**Conceptualization:** Hitoshi Yoshimura.

**Investigation:** Hitoshi Yoshimura, Shinpei Matsuda.

**Resources:** Yoshiaki Imamura.

**Supervision:** Kazuo Sano.

**Writing – original draft:** Keiichi Ohta.

**Writing – review & editing:** Keiichi Ohta, Hitoshi Yoshimura, Shinpei Matsuda, Yoshiaki Imamura, Kazuo Sano.

Keiichi Ohta orcid: 0000-0002-9148-4789.

## References

[1] Goldblum JR Folpe AL Weiss SW Enzinger and Weiss's soft tissue tumors. 6th ed. 2014;Philadelphia, PA: Saunders/Elsevier, 641-690.

[2] Golledge J Fisher C Rhys-Evans PH Head and neck liposarcoma. Cancer 1995;76:1051–8. 8625207 10.1002/1097-0142(19950915)76:6<1051::aid-cncr2820760620>3.0.co;2-4

[3] Nili F Baghai F Aghai A Well-differentiated liposarcoma of the floor of the mouth: report of a rare case and review of the literature. J Oral Maxillofac Pathol 2016;20:312–5. 27601829 10.4103/0973-029X.185984 PMC4989567

[4] Nascimento A Liposarcomas/atypical lipomatous tumors of the oral cavity: a clinicopathologic study of 23 cases. Ann Diagn Pathol 2002;6:83–93. 12004355 10.1053/adpa.2002.32375

[5] Fanburg-Smith JC Furlong MA Childers ELB Liposarcoma of the oral and salivary gland region: a clinicopathologic study of 18 cases with emphasis on specific sites, morphologic subtypes, and clinical outcome. Mod Pathol 2002;15:1020–31. 12379747 10.1097/01.MP.0000027625.79334.F5

[6] Piperi E Tosios KI Nikitakis NG Well-differentiated liposarcoma/atypical lipomatous tumor of the oral cavity: report of three cases and review of the literature. Head Neck Pathol 2012;6:354–63. 22234501 10.1007/s12105-011-0327-2 PMC3422587

[7] Cheng J Wang Y Cheng A Primary liposarcoma in oral and maxillofacial region. J Craniofac Surg 2011;22:1765–71. 21959428 10.1097/SCS.0b013e31822e626a

[8] Nikitakis NG Lopes MA Pazoki AE MDM2+/CDK4+/p53+ oral liposarcoma: case report and review of the literature. Oral Surg Oral Med Oral Pathol Oral Radiol Endod 2001;92:194–201. 11505267 10.1067/moe.2001.116815

[9] Gagari E Kabani S Gallagher GT Intraoral liposarcoma: case report and review of the literature. Oral Surg Oral Med Oral Pathol Oral Radiol and Endod 2000;89:66–72. 10630944 10.1016/s1079-2104(00)80016-5

[10] Zheng J-W Wang Y Liposarcoma in the oral and maxillofacial region: an analysis of 10 consecutive patients. J Oral Maxillofac Surg 1994;52:595–8. 8189297 10.1016/0278-2391(94)90097-3

[11] Evans HL Soule EH Winkelmann RK Atypical lipoma, atypical intramuscular lipoma, and well differentiated retroperitoneal liposarcoma: a reappraisal of 30 cases formerly classified as well differentiated liposarcoma. Cancer 1979;43:574–84. 421182 10.1002/1097-0142(197902)43:2<574::aid-cncr2820430226>3.0.co;2-7

[12] Kamikaidou N Kirita T Mishima K Liposarcoma of the cheek: report of a case. J Oral Maxillofac Surg 1998;56:662–5. 9590349 10.1016/s0278-2391(98)90469-4

[13] Allon I Vered M Dayan D Liposarcoma of the tongue: clinico-pathologic correlations of a possible underdiagnosed entity. Oral Oncol 2005;41:657–65. 16023982 10.1016/j.oraloncology.2005.01.006

[14] Kim YB Leem DH Baek JA Atypical lipomatous tumor/well-differentiated liposarcoma of the gingiva: a case report and review of literature. J Oral Maxillofac Surg 2014;72:431–9. 23992781 10.1016/j.joms.2013.06.222

[15] Saunders JR Jaques DA Casterline PF Liposarcomas of the head and neck: a review of the literature and addition of four cases. Cancer 1979;43:162–8. 367565 10.1002/1097-0142(197901)43:1<162::aid-cncr2820430124>3.0.co;2-2

[16] Sadeghi EM Sauk JJ Liposarcoma of the oral cavity. clinical, tissue culture, and ultrastructure study of a case. J Oral Pathol 1982;11:263–75. 6809914 10.1111/j.1600-0714.1982.tb00166.x

[17] Yamada K Dohara Y Nagata M A case of liposarcoma of the cheek. Jap J Clin Oncol 1979;9:123–30.

[18] Amarjit S Bhardwaj DN Nagpal BL Intraosseous liposarcoma of the maxilla and mandible: report of two cases. J Oral Surg 1979;37:593–6. 286779

[19] Suzuki H Nagayama M Kaneda T Liposarcoma of the cheek in an infant. J Oral Maxillofac Surg 1984;42:180–4. 6583363 10.1016/s0278-2391(84)80030-0

[20] Wescott WB Correll RW Multiple recurrences of a lesion at the base of the tongue. J Am Dental Assoc 1984;108:231–2. 10.14219/jada.archive.1984.0450 6584498

[21] Watanabe I Satoh M Yamane G Liposarcoma arising from the cheek of the aged: report of a case and review of the literature. Gerodontics 1985;1:148–51. 3861448

[22] Hanada M Tokuda R Ohnishi Y Benign lipoblastoma and liposarcoma in children. Acta Pathol Jpn 1986;36:605–12. 3728015 10.1111/j.1440-1827.1986.tb01050.x

[23] Dominguez FV Guglielmotti MB Flores MC Myxoid liposarcoma of the cheek. J Oral Maxillofac Surg 1990;48:395–7. 2179494 10.1016/0278-2391(90)90438-8

[24] Eidinger G Katsikeris N Gullane P Liposarcoma: report of a case and review of the literature. J Oral Maxillofac Surg 1990;48:984–8. 2203887 10.1016/0278-2391(90)90015-t

[25] Charnock DR Jett T Heise G Liposarcoma arising in the cheek: report of a case and review of the literature. J Oral Maxillofac Surg 1991;49:298–300. 1995820 10.1016/0278-2391(91)90225-b

[26] Azaz B Casap N Myxoid liposarcoma of the buccal vestibule. A case report. Int J Oral Maxillofac Surg 1991;20:308–9. 1761886 10.1016/s0901-5027(05)80162-6

[27] Guest PG Liposarcoma of the tongue: a case report and review of the literature. Br J Oral Maxillofac Surg 1992;30:268–9. 1510904 10.1016/0266-4356(92)90273-l

[28] La Quaglia MP Spiro SA Ghavimi F Liposarcoma in patients younger than or equal to 22 years of age. Cancer 1993;72:3114–9. 8221578 10.1002/1097-0142(19931115)72:10<3114::aid-cncr2820721037>3.0.co;2-i

[29] Ruacan S Onerci M Gedikoglu G Liposarcoma of the cheek: report of a case. J Oral Pathol Med 1993;22:46–7. 8419633 10.1111/j.1600-0714.1993.tb00119.x

[30] Nakahara H Shirasuna K Terada K Liposarcoma of the floor of the mouth: a case report. J Oral Maxillofac Surg 1994;52:1322–4. 7965338 10.1016/0278-2391(94)90057-4

[31] Minic AJ Liposarcomas of the oral tissues: a clinicopathologic study of four tumors. J Oral Pathol Med 1995;24:180–4. 7783007 10.1111/j.1600-0714.1995.tb01162.x

[32] Saddik M Oldring DJ Mourad WA Liposarcoma of the base of tongue and tonsillar fossa: a possibly underdiagnosed neoplasm. Arch Pathol Lab Med 1996;120:292–5. 8629909

[33] Senyuva C Yücel A Okur I A well-differentiated giant liposarcoma originating from the buccal fat pad. Ann Plast Surg 1996;37:439–43. 8905056 10.1097/00000637-199610000-00017

[34] Govender D Pillay P Primary myxoid liposarcoma with rhabdomyoblastic differentiation. Arch Pathol Lab Med 1998;122:740–2. 9701338

[35] Nelson W Chuprevich T Galbraith DA Enlarging tongue mass. J Oral Maxillofac Surg 1998;56:224–7. 9461149 10.1016/s0278-2391(98)90873-4

[36] Hasegawa H Kawakami T Eda S Mixed-type liposarcoma of the oral cavity: a case with unusual features and a long survival. J Oral Pathol Med 1999;28:141–4. 10069544 10.1111/j.1600-0714.1999.tb02013.x

[37] Piattelli A Di Alberti L Favia GF Liposarcoma involving the periodontal tissues. A case report. J Periodontol 2000;71:322–4. 10711624 10.1902/jop.2000.71.2.322

[38] Orita Y Nishizaki K Ogawara T Liposarcoma of the tongue: case report and literature update. Ann Otol Rhinol Laryngol 2000;109:683–6. 10903052 10.1177/000348940010900713

[39] Moore PL Goede A Phillips DE Atypical lipoma of the tongue. J Laryngol Otol 2001;115:859–61. 11668010 10.1258/0022215011909198

[40] Bengezi OA Kearns R Shuhaibar H Myxoid liposarcoma of the tongue. J Otolaryngol 2002;31:327–8. 12512900 10.2310/7070.2002.29966

[41] Nunes FD Loducca SVL de Oliveira EMF Well-differentiated liposarcoma of the tongue. Oral Oncol 2002;38:117–9. 11755832 10.1016/s1368-8375(01)00030-6

[42] Angeles RM Vasquez J Kim O Pathologic quiz case: an 86-year-old man with a painless right tongue mass. Atypical lipomatous tumor of the tongue. Arch Pathol Lab Med 2005;129:253–4. 15679435 10.5858/2005-129-253-PQCAYM

[43] DeWitt J Heidelman J Summerlin DJ Atypical lipomatous tumors of the oral cavity: a report of 2 cases. J Oral Maxillofac Surg 2008;66:366–9. 18201625 10.1016/j.joms.2006.10.035

[44] Tanaka N Mimura M Kimijima Y Ultrastructure of oral sarcoma. Med Electron Microsc 2002;35:204–16. 12658355 10.1007/s007950200024

[45] Friedman JL Bistritz JI Robinson MJ Pleomorphic liposarcoma of the pterygomandibular space involving the maxilla. Oral Surg Oral Med Oral Pathol Oral Radiol Endod 1995;79:488–91. 7614211 10.1016/s1079-2104(05)80133-7

[46] Favia G Maiorano E Orsini G Myxoid liposarcoma of the oral cavity with involvement of the periodontal tissues. J Clin Periodontol 2001;28:109–12. 11168734 10.1034/j.1600-051x.2001.028002109.x

[47] Hattori H Atypical lipomatous tumor of the lip with pleomorphic lipoma-like myxoid area, clinically simulating mucocele. J Oral Pathol Med 2002;31:561–4. 12269996 10.1034/j.1600-0714.2002.00151.x

[48] Fusetti M Silvagni L Eibenstein A Myxoid liposarcoma of the oral cavity: case report and review of the literature. Acta Otolaryngol 2001;121:759–62. 11678177 10.1080/00016480152583728

[49] Yamaguchi S Nagasawa H Suzuki T Sarcomas of the oral and maxillofacial region: a review of 32 cases in 25 years. Clin Oral Investig 2004;8:52–5. 10.1007/s00784-003-0233-4 15281217

[50] Capodiferro S Scully C Maiorano E Liposarcoma circumscriptum (lipoma-like) of the tongue: report of a case. Oral Dis 2004;10:398–400. 15533218 10.1111/j.1601-0825.2004.01040.x

[51] da Cunha IW Kowalski LP Soares FA Dedifferentiated liposarcoma of the oral cavity with angiosarcomatous dedifferentiation. Virchows Archiv 2005;446:456–9. 15806379 10.1007/s00428-005-1207-5

[52] Kacker A Taskin M Atypical intramuscular lipoma of the tongue. J Laryngol Otol 1996;110:189–91. 8729513 10.1017/s0022215100133146

[53] Angiero F Sidoni A Stefani M Liposarcoma of the oral cavity--case reports of the pleomorphic and the dedifferentiated variants and a review of the literature. Anticancer Res 2006;26:4857–67. 17214352

[54] Chan WY McHenry IDS Carter LM Gingival liposarcoma: an unusual polyp. Br J Oral Maxillofac Surg 2008;46:150–1. 17284338 10.1016/j.bjoms.2006.12.006

[55] Sobral APV de Oliveira Lima DN Cazal C Myxoid liposarcoma of the lip: correlation of histological and cytological features and review of the literature. J Oral Maxillofac Surg 2007;65:1660–4. 17656299 10.1016/j.joms.2006.06.264

[56] McElderry J McKenney JK Stack BC High-grade liposarcoma metastatic to the gingival mucosa: case report and literature review. Am J Otolaryngol 2008;29:130–4. 18314026 10.1016/j.amjoto.2007.04.001

[57] Laco J Mentzel T Hornychova H Atypical lipomatous tumors of the tongue: report of six cases. Virchows Archiv 2009;455:383–8. 19816710 10.1007/s00428-009-0835-6

[58] Moritani N Yamada T Mizobuchi K Atypical lipomatous tumor of the tongue: report of a case. Acta Med Okayama 2010;64:257–61. 20802543 10.18926/AMO/40134

[59] Paragis Sanchez T Bannwart C Murilo Araújo D Well-differentiated liposarcoma of the tongue. A case report. Minerva Stomatol 2008;57:383–7. 18784638

[60] Cheng J Yu H Wang L Primary oral and maxillofacial liposarcoma: a clinicopathological and immunohistochemical study of eleven cases. Arch Med Sci 2012;2:316–23. 10.5114/aoms.2012.28560 PMC3361045 22662006

[61] Nichols EM Mirmiran A Garofalo MC Recurrent myxoid liposarcoma of the buccal mucosa in a young boy: a case report and review of the literature. Ear Nose Throat J 2011;90:e27–31. 10.1177/014556131109001215 22180120

[62] Sugawara C Takahashi A Kawano F Intraoral ultrasonography of tongue mass lesions. Dentomaxillofac Radiol 2016;45:20150362. 26915405 10.1259/dmfr.20150362 PMC5084697

[63] Nimura F Nakasone T Matsumoto H Dedifferentiated liposarcoma of the oral floor: a case study and literature review of 50 cases of head and neck neoplasm. Oncol Lett 2018;15:7681–8. 29740489 10.3892/ol.2018.8274 PMC5934721

[64] Kaczmarczyk D Jesionek-Kupnicka D Kubiak M Atypical lipomatous tumor of the cheek – a case report. Otolaryngol Pol 2013;67:218–21. 23911052 10.1016/j.otpol.2012.06.022

[65] Dubin MR Chang EW Liposarcoma of the tongue: case report and review of the literature. Head Face Med 2006;2:21. 16872488 10.1186/1746-160X-2-21 PMC1553437

[66] Miyazaki M Aoki M Oba S A rare case of dedifferentiated liposarcoma of the sinonasal cavity: a case report. Mol Clin Oncol 2017;7:539–42. 29046788 10.3892/mco.2017.1379 PMC5639279

[67] Sheffield BS Nielsen TO Myxoid liposarcoma in a 91-year-old patient. Mol Cytogenet 2013;6:50. 24252207 10.1186/1755-8166-6-50 PMC3843574

[68] Kale HA Prabhu AV Sinelnikov A Fat: friend or foe? A review of fat-containing masses within the head and neck. Br J Radiol 2016;89:20150811. 27542075 10.1259/bjr.20150811 PMC5124824

[69] Razek AA Huang BY Soft tissue tumors of the head and neck: imaging-based review of the WHO classification. Radiographics 2011;31:1923–54. 22084180 10.1148/rg.317115095

[70] Ishii J Nagasawa H Wadamori T Ultrasonography in the diagnosis of palatal tumors. Oral Surg Oral Med Oral Pathol Oral Radiol Endod 1999;87:39–43. 9927078 10.1016/s1079-2104(99)70292-1

[71] Charest M Hickeson M Lisbona R FDG PET/CT imaging in primary osseous and soft tissue sarcomas: a retrospective review of 212 cases. Eur J Nucl Med Mol Imaging 2009;36:1944–51. 19593561 10.1007/s00259-009-1203-0

[72] Suzuki R Yanagawa T Sato J PET evaluation of fatty tumors in the extremity: possibility of using the standardized uptake value (SUV) to differentiate benign tumors from liposarcoma. Ann Nucl Med 2005;19:661–70. 16444991 10.1007/BF02985114

[73] Hisaoka M Lipoblast: morphologic features and diagnostic value. J UOEH 2014;36:115–21. 24930875 10.7888/juoeh.36.115

[74] Stojanov IJ Mariño-Enriquez A Bahri N Lipomas of the oral cavity: utility of MDM2 and CDK4 in avoiding overdiagnosis as atypical lipomatous tumor. Head Neck Pathol 2019;13:169–76. 29748845 10.1007/s12105-018-0928-0 PMC6513928

[75] Dei Tos AP Doglioni C Piccinin S Coordinated expression and amplification of the MDM2,CDK4, and HMGI-C genes in atypical lipomatous tumours. J Pathol 2000;190:531–6. 10727978 10.1002/(SICI)1096-9896(200004)190:5<531::AID-PATH579>3.0.CO;2-W

[76] Nakayama T Toguchida J Wadayama B-I MDM2 gene amplification in bone and soft-tissue tumors: association with tumor progression in differentiated adipose-tissue tumors. Int J Cancer 1995;64:342–6. 7591308 10.1002/ijc.2910640511

[77] Thway K Flora R Shah C Diagnostic utility of p16, CDK4, and MDM2 as an immunohistochemical panel in distinguishing well-differentiated and dedifferentiated liposarcomas from other adipocytic tumors. Am J Surg Pathol 2012;36:462–9. 22301498 10.1097/PAS.0b013e3182417330

[78] Kammerer-Jacquet S-F Thierry S Cabillic F Differential diagnosis of atypical lipomatous tumor/well-differentiated liposarcoma and dedifferentiated liposarcoma: utility of p16 in combination with MDM2 and CDK4 immunohistochemistry. Hum Pathol 2017;59:34–40. 27597521 10.1016/j.humpath.2016.08.009

[79] Davis EC Ballo MT Luna MA Liposarcoma of the head and neck: the university of texas M. D. Anderson cancer center experience. Head Neck 2009;31:28–36. 18767171 10.1002/hed.20923

[80] Gritli S Khamassi K Lachkhem A Head and neck liposarcomas. Auris Nasus Larynx 2010;37:347–51. 19857936 10.1016/j.anl.2009.08.003

[81] McCulloch TM Makielski KH McNutt MA Head and neck liposarcoma: a histopathologic reevaluation of reported cases. Arch Otolaryngol Head Neck Surg 1992;118:1045–9. 1389054 10.1001/archotol.1992.01880100035010

[82] Zhu H Sun J Wei S Well-differentiated laryngeal/hypopharyngeal liposarcoma in the MDM2 era report of three cases and literature review. Head Neck Pathol 2017;11:146–51. 27492446 10.1007/s12105-016-0747-0 PMC5429270

[83] Patel SR Burgess MA Plager C Myxoid liposarcoma. experience with chemotherapy. Cancer 1994;74:1265–9. 8055448 10.1002/1097-0142(19940815)74:4<1265::aid-cncr2820740414>3.0.co;2-x

[84] Jones RL Fisher C Al-Muderis O Differential sensitivity of liposarcoma subtypes to chemotherapy. Eur J Cancer 2005;41:2853–60. 16289617 10.1016/j.ejca.2005.07.023

